# Transient Non-Fourier Fracture Analysis of a Central Crack in Aluminium Foams by Guyer–Krumhansl Theory

**DOI:** 10.3390/ma19142933

**Published:** 2026-07-08

**Authors:** Kunyang Cao, Jiaqi Zong, Zhijun Liu, Zengtao Chen, Wenzhi Yang

**Affiliations:** 1 School of Intelligent Manufacturing, Henan Polytechnic, Zhengzhou 450046, China; 37006@hnzj.edu.cn; 2 CCCC Mechanical & Electrical Engineering Co., Ltd., Beijing 101300, China; zongjq007@163.com; 3 College of Civil Engineering and Mechanics, Lanzhou University, Lanzhou 730000, China; liuzhijun@lzu.edu.cn; 4 Department of Mechanical Engineering, University of Alberta, Edmonton, AB T6G 1H9, Canada; zengtao@ualberta.ca

**Keywords:** Guyer–Krumhansl theory, central crack, stress intensity factor, Laplace transform, aluminium foam

## Abstract

The measured temperature–time history of aluminium foams in heat pulse experiments has demonstrated that Fourier’s law fails while the Guyer–Krumhansl (G-K) theory performs satisfactorily in characterising their thermal transport process. The purpose of this work is to explore the non-Fourier heat transport and fracture behaviours of an aluminium foam plate containing a central crack subjected to sudden hot thermal shocks by utilising the G-K model. The weight function methodology coupled with the superposition principle is adopted to address the mode-I fracture problem. Parametric investigations are conducted to examine the influences of thermal relaxation time, nonlocal length, and relative density of aluminium foams on the transient heat transfer and the dynamic fracture responses. The time lagging effect and the thermal nonlocal length scale are demonstrated to be critical factors governing the transient processes. The findings will facilitate an in-depth understanding and promote aluminium foam’s applications as thermal insulation materials in extreme thermal conditions.

## 1. Introduction

As a new type of advanced architected cellular materials, aluminium foams have attracted unprecedented attention in material science and diverse engineering applications, like automobile, microelectromechanical systems, and aerospace industries [1,2,3,4,5]. Characterised by a three-dimensional network of numerous interconnected gas pores dispersed within the aluminium matrix, aluminium foams usually exhibit an extraordinary combination of mechanical and thermal properties compared to conventional solid metal materials. Unlike traditional lightweight materials such as polymers or polymer-based composites, aluminium foams retain the intrinsic advantages of metallic materials, including excellent thermal and electric conductivities, high melting point and mechanical strength, while simultaneously possessing an ultralow density, exceptional specific strength, excellent sound insulation and an outstanding energy absorption capacity.

Distinguished by these extraordinary physical properties, aluminium foams have emerged as indispensable materials in diverse thermal environments. For example, during hypersonic flight, the vehicle surfaces are exposed to short-duration and ultrahigh temperatures, where conventional metallic materials fail catastrophically under such extreme thermal shock conditions. In contrast, the closed-cell aluminium foams perform satisfactorily as effective thermal insulation fillers since their thermal conductivities are as low as 1/50 that of bulk aluminium. In addition, open cell aluminium foams effectively enhance the heat dissipation in the active cooling systems owing to their superior surface-area-to-volume ratios. To ensure their reliable performance in various thermal conditions, numerous studies have focused on the heat transfer process. Bhattacharya and Mahajan [6] conducted experiments to systematically investigate the buoyancy-induced convection characteristics of aluminium foams with diverse pore densities and porosities. They noticed the considerable enhancement of the heat transfer coefficients in the heat sinks and then proposed a new empirical relation to estimate the Nusselt number. Mancin et al. [7] experimentally examined the air forced thermal convection and pressure drop characteristics of aluminium foams under various numbers of pores per linear inch. The influence of pore density, foam core height and imposed heat flux on the heat transfer process was considered. From experimental results, they established two universal correlations for thermal coefficient and pressure drop estimations. Feng et al. [8] numerically revealed the convection melting heat transfer characteristics in aluminium foams under sinusoidal thermal boundary conditions, and elucidated the effects of porosity, temperature amplitude and frequency on melting rate and temperature field distribution. Lin et al. [9] compared the applicability of locally thermal equilibrium and non-equilibrium models for convective heat transfer in aluminium foams by a computational fluid dynamics method and provided the critical flow velocity and porosity ranges for each model. Singh et al. [10] combined experimental and numerical methods to explore the forced convection thermal characteristics of high-porosity aluminium foams under jet array impingement in channel flow and found that the combination of jet arrays and aluminium foams could increase the heat transfer coefficient by 26–48% compared with pure channel flow.

Despite significant advancements in the heat transfer problem of aluminium foams ranging from experimental investigations to theoretical and numerical studies, one critical challenge remains unsolved; that is, most studies were established within the framework of classical Fourier’s law, which assumes thermal energy is capable of propagating instantaneously through solids with infinite speed. Although this approximation performs well for most conventional applications, significant deviations take place between experimental measurements and theoretical calculations when the physical process’s characteristic time approaches the order of the heat propagation time scales [11]. Specifically, a noticeable non-Fourier effect takes place in the porous material compared to the bulk materials since thermal transport within these discrete structures requires a longer transient period to attain local thermal equilibrium between the solid and the gaseous phases. Furthermore, aluminium foams typically operate under extremely high temperatures with severe thermal gradients, where the classical Fourier theory is no longer applicable. Therefore, an accurate description of thermal transport in aluminium foams is indispensable for promoting aluminium foams’ applications as thermal insulation materials in extreme thermal conditions.

To address the fundamental limitation of the classical theory, several non-Fourier methodologies were proposed. The most straightforward approach characterising the non-Fourier effect is the Cattaneo–Vernotte (C-V) theory [12,13], which introduces a lagging time between the temperature gradients and the corresponding heat flux response. Subsequently, Tzou proposed the dual-phase-lag (DPL) theory by introducing an additional thermal relaxation time to take the microstructural interaction between the phonons and electrons into account [11]. Furthermore, as the nonlocal extension of the C-V equation, the G-K model represents a more comprehensive approach of modelling non-classical heat conduction regimes, as it uniquely integrates Fourier diffusion, thermal wave, and ballistic transport behaviours in the nanoscales into a unified constitutive formulation [14]. According to the experiments of heat conduction in porous sands [11], the C-V constitutive equation has been demonstrated to be incapable of predicting the complex heat transfer characteristics in these discrete materials. On the other hand, recent heat pulse experiments by A. Fehér and R. Kovács [15,16] have confirmed that the G-K theory precisely captures the transient temperature changes in porous materials, like carbon foams and aluminium foams. Considering the widespread use of aluminium foams in harsh high-temperature engineering scenarios, studying their heat transfer, thermal deformation, and thermal fracture behaviours based on the G-K model is critical for ensuring structural safety. To this end, this paper systematically examines the thermoelastic and thermal fracture characteristics of porous aluminium foams under the non-Fourier G-K heat conduction framework.

## 2. Problem Description

**Figure 1:**
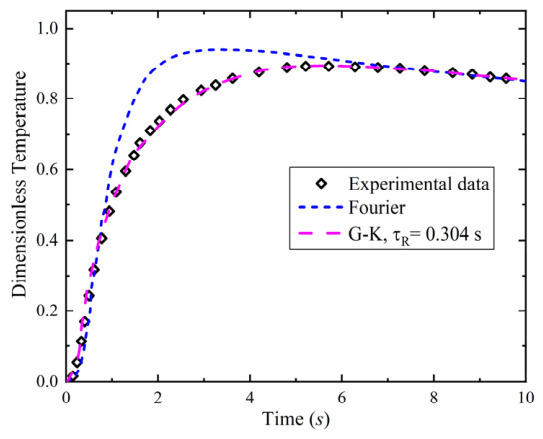
The experimentally measured dimensionless temperature history of aluminium foam with comparison to predictions of the Fourier model and G-K theory [15,16].

The traditional Fourier model usually breaks down in thermal management systems within micro/nano spatial or temporal scales, since the errors caused by its implications of infinite heat spread speed become noticeable in these cases. In addition, experimental measurements in porous sand, composite materials, carbon foams, and aluminium foams suggest the failure of Fourier’s law in these materials even on macroscopic scales at room temperature. According to the findings of A. Fehér and R. Kovács [15,16], the Guyer–Krumhansl theory offers a precise macroscopic formulation of the temperature history of these porous materials, which can be expressed by:

(1) $$q+\tau_{R}\frac{\partial q}{\partial t}+\lambda \nabla T=\chi^{2}\left[\nabla^{2}q+2\nabla (\nabla \cdot q)\right]$$

where q denotes the heat flux vector and T is the temperature, λ denotes the thermal conductivity, $\tau_{R}$ represents the thermal relaxation time and χ is the thermal nonlocal parameter. The introduction of the thermal relaxation time and nonlocal parameter provides the G-K model’s capability of characterising the non-Fourier effect in the heating process of aluminium foams, where the experimental measured temperature history of aluminium foam with comparison to predictions of the Fourier model and G-K theory is displayed in Figure 1. It is noticeable that the thermal relaxation time reaches up to 0.304 s, which is significantly higher than the counterpart values in pure metals (usually in picoseconds or femtoseconds). This finding highlights the necessity of exploring the non-Fourier thermal, elastic, and fracture behaviours of aluminium foams even at conventional thermophysical applications.

**Figure 2:**
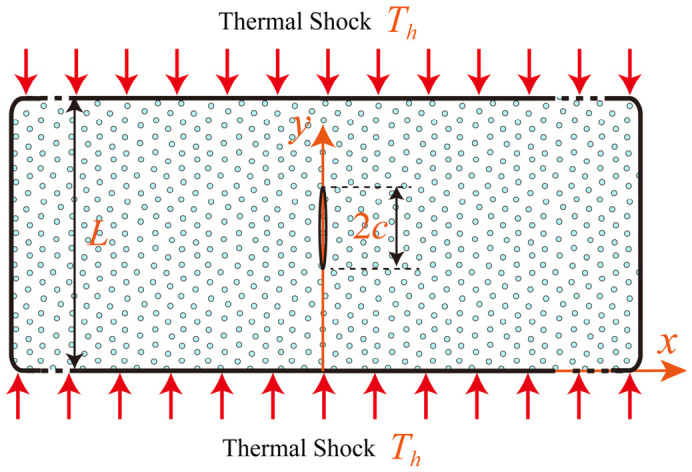
A porous aluminium foam plate of thickness *L* subjected to abrupt hot thermal shocks on its bounding surfaces.

To advance our knowledge of aluminium foams working as thermal insulation materials in the high-temperature application, this article considers a plate with thickness *L* constructed by porous aluminium foams subjected to hot thermal shocks on both the top and bottom bounding surfaces, as presented in Figure 2. Assume a centre crack of length 2*c* exists in the aluminium foam plate. The whole plate is initially at ambient temperature $T_{r}$. At t=0, the abrupt hot thermal shocks $T_{h}$ are exerted on the two surfaces, so the thermal boundary conditions can be written as:

(2) $$T(y,t)=T_{h}H(t),\;\;\;(y=0,\;L)$$

where H(t) denotes the Heaviside function. Since the centre crack is parallel to the heat flow direction, the thermal transfer process in the plate is one-dimensional. For simplicity, a plane-stress assumption is adopted in this analysis. No external mechanical loading is applied on the borders, so the mechanical boundary conditions are written as:

(3) $$\sigma_{yy}(0,t)=\sigma_{yy}(L,t)=0$$

(4) $$\sigma_{xy}(0,t)=\sigma_{xy}(L,t)=0$$

The reason behind the wide applications of aluminium foams as thermal insulation materials is their lightweight advantage to reduce the weight of engineering structures as well as guarantee the strength. Compared to the aluminium solid metal, numerous interstitial gas pores exist in the aluminium foams, which is exactly the root cause of their prominent non-Fourier thermal effect observed in the heat pulse experiments. The heat spreads firstly along the solid framework, and then it takes a finite time for the aluminium foams to reach the locally thermal equilibrium. To define the capability of reducing the weight of the aluminium foams, the relative density is introduced as:

(5) $$\rho_{r}=\frac{\rho_{eff}}{\rho_{A}},$$

where $\rho_{eff}$ is the effective density of the aluminium foam and $\rho_{A}$ represents the density of the dense aluminium. Aluminium foams are discrete materials whose thermomechanical properties exhibit spatial discontinuity throughout their interior structures. Accordingly, the effective material properties would be beneficial in theoretically modelling their heat transfer, thermal stress, and fracture problems. In accordance with the rule of mixtures, the effective heat capacity of aluminium foams is determined by the arithmetic average of the aluminium metal and the filled gas weighed by their respective volume fractions [17]:

(6) $$\rho_{eff}e_{eff}=(\rho_{A}e_{A})\rho_{r}+(\rho_{g}e_{g})(1-\rho_{r})$$

where $e_{eff}$ is the effective specific heat, and the subscripts “*A*” and “*g*” denote the respective densities and specific heats of the aluminium and filled gas. According to [17], the aluminium foam’s effective thermal conductivity is calculated via:

(7) $$\lambda_{eff}=\frac{1}{3}(\rho_{r}+2\rho_{r}^{\;\;3/2})\lambda_{A}+(1-\rho_{r})\lambda_{g},$$

where $\lambda_{A}$ and $\lambda_{g}$ denote the respective thermal conductivities of the aluminium and filled gas. In addition, based on the classical cellular solids theory proposed by Gibson and Ashby [18], the aluminium foam’s effective Young’s modulus $E_{eff}$ and shear modulus $G_{eff}$ can be estimated by the following expression:

(8) $$E_{eff}=\rho_{r}^{\;\;2}E_{A},\;\;\;G_{eff}=\frac{3}{8}\rho_{r}^{\;\;2}G_{A}$$

where $E_{A}$ and $G_{A}$ represent the bulk aluminium’s Young’s modulus and shear modulus. Furthermore, the open cell metallic foam’s elastic response is exclusively governed by the expansion of the metallic solid framework, since the gas filled in the pores contributes little to the thermal deformation. Consequently, the coefficient of thermal expansion of aluminium foam is identical to that of the corresponding dense aluminium. By employing these effective thermomechanical properties of aluminium foams, the following section presents a theoretical modelling framework on the basis of the G-K model, which is dedicated to analysing the non-Fourier effect on heat transfer characteristics, mechanical deformation and fracture behaviours of porous aluminium foams. It should be acknowledged that the effective properties used in this work are based on homogenised isotropic relations, which treat the aluminium foam as a continuum with average thermomechanical characteristics. In reality, foams are heterogeneous, often anisotropic, and their pore-scale stress and temperature fields can deviate substantially from the macroscopic averages. The present homogeneous description therefore provides an estimate of the global heat transfer, thermal stress, and fracture driving force, but it does not resolve local stress concentrations near individual pores that may trigger micro-crack initiation. Consequently, the predicted variables should be interpreted as a macroscopic engineering indicator, and the trends revealed by the parametric study are expected to remain qualitatively valid for realistic foams, but quantitative accuracy would require more refined modelling.

## 3. Theoretical Solutions

In order to derive the thermal governing equation that describes the heat transfer process in aluminium foams, the G-K model, whose mathematical expression is presented in Equation (1) and considers the non-Fourier heat transfer characteristics of porous metallic materials, needs to be integrated into the fundamental principle of thermal energy conservation:

(9) $$-\nabla \cdot q=\rho e_{eff}\frac{\partial T}{\partial t}.$$

The governing equation characterising the heating process in porous aluminium foams can be derived as:

(10) $$(1+\tau_{R}\frac{\partial }{\partial t})\frac{\partial T}{\partial t}=\frac{\lambda }{\rho e_{eff}}\frac{\partial^{2}T}{\partial y^{2}}+3\chi^{2}\frac{\partial }{\partial t}\frac{\partial^{2}T}{\partial y^{2}}$$

To simplify the subsequent analysis, we define the dimensionless quantities as follows:

(11) $$(\bar{x},\bar{y},\bar{c},\bar{L},\bar{\chi })=\frac{\left(x,y,c,L,\chi \right)}{\Gamma },\;\theta =\frac{T-T_{0}}{T_{h}-T_{0}},\;(\bar{t},{\bar{\tau }}_{R})=\frac{10(t,\tau_{R})}{\Gamma^{2}/\kappa_{A}},$$

where $\;\kappa_{A}=\lambda_{A}/(\rho_{A}e_{A})$ is the dense aluminium’s thermal diffusivity, Γ represents the characteristic length, θ denotes the non-dimensional temperature. Then, we obtain the dimensionless governing equation as follows:

(12) $$(1+{\bar{\tau }}_{R}\frac{\partial }{\partial \bar{t}})\frac{\partial \theta }{\partial \bar{t}}=(\frac{\kappa_{eff}}{10\kappa_{A}}+3{\bar{\chi }}^{2}\frac{\partial }{\partial \bar{t}})\frac{\partial^{2}\theta }{\partial {\bar{y}}^{2}}$$

where the foam’s effective thermal diffusivity is $\;\kappa_{eff}=\lambda_{eff}/(\rho_{eff}e_{eff})$. The thermal boundary conditions given in Equation (2) thus reduce to:

(13) $$\theta (\bar{y},\bar{t})=H(\bar{t}),\;\;(\bar{y}=0,\bar{L})$$

We employ the Laplace transform method in this investigation to solve the partial differential Equation (12) associated with the boundary conditions specified in Equation (13). After carrying out the Laplace transform, the corresponding equation in the Laplace domain is:

(14) $$\frac{d^{2}\theta }{d{\bar{y}}^{2}}=\frac{(1+{\bar{\tau }}_{R}m)m}{\left(\frac{\kappa_{eff}}{10\kappa_{A}}+3{\bar{\chi }}^{2}m\right)}\theta$$

where *m* denotes the Laplace transform variable. Accordingly, the boundary condition of Equation (13) in the Laplace domain becomes:

(15) $$\theta (\bar{y},m)=\frac{1}{m},\;\;\;(\bar{y}=0,\bar{L}).$$

The general solution of the ordinary differential Equation (14) takes the form:

(16) $$\theta (\bar{y},m)=R_{a}(m)e^{-\delta \bar{y}}+R_{b}(m)e^{\delta \bar{y}}$$

where $\delta =\sqrt{\left(1+{\bar{\tau }}_{R}m)m/(\frac{\kappa_{eff}}{10\kappa_{A}}+3{\bar{\chi }}^{2}m\right)}$. $R_{a}(m)$ and $R_{b}(m)$ are unknown terms, which can be determined by the boundary conditions.

Inserting the relations given in Equation (16) into Equation (15) leads to:

(17) $$R_{a}(m)=\frac{1-e^{-\delta \bar{L}}}{m(1-e^{-2\delta \bar{L}})},R_{b}(m)=\frac{e^{-\delta \bar{L}}-e^{-2\delta \bar{L}}}{m(1-e^{-2\delta \bar{L}})}$$

Thus far, the temperature field of porous aluminium foams in the Laplace domain has been determined.

To investigate the deformation and fracture behaviours of aluminium foams driven by thermal expansion, this work assumes the problem to be in a quasi-static, uncoupled thermoelastic state under plane-stress conditions for the sake of brevity and computational simplicity. The central crack presented in Figure 2 is parallel to the heat flow direction; therefore, the temperatures, thermal stresses and strains depend solely on the *y* coordinate, and all shear stress and shear strain components vanish within this theoretical model. According to the compatibility condition:

(18) $$\frac{\partial^{2}\varepsilon_{xx}}{\partial y^{2}}+\frac{\partial^{2}\varepsilon_{yy}}{\partial x^{2}}=\frac{\partial^{2}\gamma_{xy}}{\partial x\partial y}$$

we have:

(19) $$\frac{\partial^{2}\varepsilon_{xx}}{\partial y^{2}}=0$$

Then

(20) $$\varepsilon_{xx}=C_{1}y+C_{2}$$

where $C_{1},\;C_{2}$ are constants. As illustrated in Figure 2, the thermal shock loadings are symmetric, so the plate would not bend and $C_{1}=0$. Meanwhile, based on the equilibrium equation and the traction free boundary conditions on the two surfaces, it is convenient to find $\sigma_{yy}=0$ for the present problem. By utilising Hook’s law, we have:

(21) $$\sigma_{xx}(y,t)=E_{eff}\left[\varepsilon_{xx}-\alpha \cdot (T-T_{0})\right]$$

The unstrained plate undergoes free thermal expansion without any external axial force. Hence, the thermal stress components satisfy the corresponding relational equation:

(22) $$\int_{0}^{L}\sigma_{xx}dy=0.$$

Consequently, one can derive:

(23) $$\varepsilon_{xx}=\frac{\alpha }{L}\int_{0}^{L}\left(T-T_{0}\right)dy.$$

The thermal stress takes the following form:

(24) $$\sigma_{xx}(y,t)=E_{eff}\alpha \left(\frac{1}{L}\int_{0}^{L}\left(T-T_{0}\right)dy-(T-T_{0})\right).$$

where $E_{eff}$ is the aluminium foam’s effective Young’s modulus and α is the coefficient of thermal expansion of aluminium foam. In order to facilitate subsequent derivation and keep it consistent with the variable settings of Equation (11), the thermal stress is expressed in a normalised form:

(25) $${\bar{\sigma }}_{xx}=\frac{\sigma_{xx}}{10^{-3}E_{A}\alpha_{A}(T_{h}-T_{0})}$$

Combining Equations (16), (24) and (25), one can derive:

(26) $${\bar{\sigma }}_{xx}(\bar{y},m)=10^{3}\rho_{r}^{\;\;2}\left(\frac{R_{a}}{\delta \bar{L}}(1-e^{-\delta \bar{L}})+\frac{R_{b}}{\delta \bar{L}}(e^{\delta \bar{L}}-1)-R_{a}e^{-\delta \bar{y}}-R_{b}e^{\delta \bar{y}}\right)$$

where $\rho_{r}$ is the relative density. At this stage, the non-dimensional thermal stresses are fully obtained. Afterwards, the superposition method is adopted to analyse the centre problem by exerting transient thermal stress on the central crack faces. The mode-I SIF of the central crack in the aluminium foam plate is then assessed via the weight function approach. For a central crack in a finite strip under symmetric concentrated force *P* applied at a distance *b* from the crack centre on the crack faces [19], the SIF is:

(27) $$K_{I}=\frac{2P}{\sqrt{L}}\Psi (\frac{c}{L/2},\frac{b}{c})$$

where L is the thickness and c is the crack half length. The correction function Ψ is given as:

(28) $$\begin{array}{l} \Psi (\frac{c}{L/2},\frac{b}{c})=\frac{\left\{1+0.297\sqrt{1-{\left(\frac{b}{c}\right)}^{2}}\left(1-\cos\frac{\pi c}{L}\right)\right\}\cdot \sqrt{\tan\frac{\pi c}{L}}}{\sqrt{1-{\left(\cos\frac{\pi c}{L}/\cos\frac{\pi b}{L}\right)}^{2}}} \end{array}$$

Substituting the distributed thermal stress from Equation (26), the dimensionless stress intensity factor in the Laplace domain is obtained via the superposition principle, yielding:

(29) $${\bar{K}}_{I}(s)=\frac{2}{\sqrt{\bar{L}}}\int_{\bar{L}/2}^{\bar{L}/2+\bar{c}}\Psi (\frac{\bar{c}}{\bar{L}/2},\frac{\bar{y}-\bar{L}/2}{\bar{c}}){\bar{\sigma }}_{xx}(\bar{y},m)d\bar{y}$$

where

(30) $$\begin{array}{l} \Psi (\frac{\bar{c}}{\bar{L}/2},\frac{\bar{y}-\bar{L}/2}{\bar{c}})=\frac{\left\{1+0.297\sqrt{1-{\left(\frac{\bar{y}-\bar{L}/2}{\bar{c}}\right)}^{2}}\left(1-\cos\frac{\pi \bar{c}}{\bar{L}}\right)\right\}\cdot \sqrt{\tan\frac{\pi \bar{c}}{\bar{L}}}}{\sqrt{1-{\left(\cos\frac{\pi \bar{c}}{\bar{L}}/\cos\frac{\pi (\bar{y}-\bar{L}/2)}{\bar{L}}\right)}^{2}}} \end{array}$$

According to Equations (11) and (25), the above SIF is normalised by:

(31) $${\bar{K}}_{I}=\frac{K_{I}}{10^{-3}E_{A}\alpha_{A}(T_{h}-T_{0})\Gamma }$$

Thus far, the dimensionless temperature variation, thermal stress field and stress intensity factor have been derived in the Laplace domain, as summarised in Equations (16), (26) and (29). To convert these formulations into explicit solutions in the physical time domain, the numerical Laplace inversion algorithm developed by Stehfest [20] is adopted throughout this work.

It is worth noting that the present analysis rests on the simplifications: one-dimensional heat conduction, plane stress, uncoupled thermoelasticity, and quasi-static mechanical response, which are jointly appropriate for a thin plate under moderate thermal shock, provided the heating time scale far exceeds the elastic wave propagation time and thermomechanical coupling is negligible. These assumptions, however, impose clear limitations: the 1-D conduction model excludes lateral edge effects and multi-dimensional heat flow; the plane-stress formulation requires revision to the plane strain for thicker plates; the uncoupled linear–elastic framework does not capture plastic dissipation; and the quasi-static treatment omits possible dynamic stress-wave overshoots under ultra-rapid heating. These considerations define the range of validity of the present study.

## 4. Numerical Results and Discussion

To convert the analytical solutions derived in the Laplace domain back to the physical time domain, we apply the corresponding numerical inversion procedure to Equations (16), (26) and (29) via the MATLAB package (R2022a). This numerical approach enables us to accurately compute the transient temperature changes, dynamical thermal stress distributions and SIFs of the porous aluminium foams. For consistent dimensionless analysis, the thickness of the plate is defined as the characteristic length scale, leading to a normalised thickness of 1 throughout the present work. The effective thermal and mechanical properties of aluminium foam are jointly determined by the solid metal and filled air, whose physical properties are summarised in Table 1. To simplify the notation, all overbars denoting dimensionless quantities are omitted. All variables appearing in this section are non-dimensional by default.

**Figure 3:**
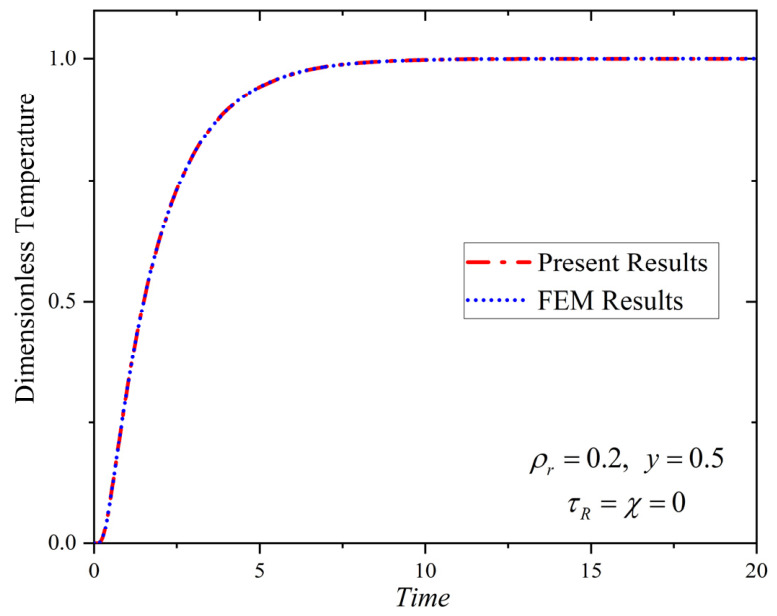
Verification of dimensionless temperature of the present results with FEM results under the Fourier heat conduction theory.

To verify our solution procedure, we first check the limiting case where both the thermal relaxation time and the nonlocal length vanish ($\tau_{R}=\chi =0$). Under these conditions the G–K model reduces exactly to the classical Fourier heat conduction law. The finite element method (FEM) is employed to build a simple one-dimensional model for Equation (12). Figure 3 compares the transient dimensionless temperature at *y* = 0.5 obtained from our solution with the FEM results. The curves coincide with each other, confirming that the Laplace inversion and the temperature solution are correctly implemented.

**Figure 4:**
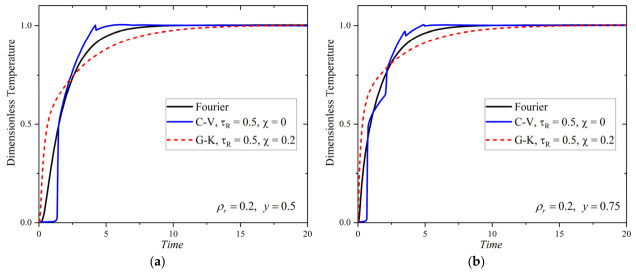
Transient dimensionless temperature for locations (**a**) y=0.5 and (**b**) y=0.75 under three heat conduction theories.

**Figure 5:**
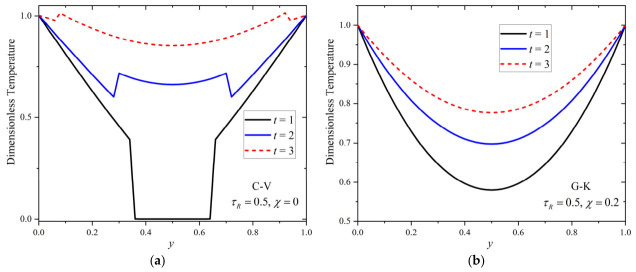
Dimensionless temperature distributions along the thickness direction for the (**a**) C-V model and (**b**) G-K model.

As discussed in the preceding theoretical part, the G-K model degenerates into the C-V model in the limit of zero nonlocal length, whereas the classical Fourier heat conduction law represents the limiting scenario where both the nonlocal length scale and the thermal relaxation time are identically zero. To initiate our parametric study, we first analyse the thermal transportation under various heat conduction frameworks. The aluminium foam plate is exposed to hot thermal shocks on both the top and bottom surfaces; therefore, the temperature distribution in the plate is symmetrical about the centre line *y* = 0.5 for the whole heating period. We pick two critical points to analyse the temperature–time history variation at *y* = 0.5 and *y* = 0.75 within three heat conduction frameworks, as illustrated in Figure 4. The Fourier’s result presents a smooth monotonic increase in the temperature of both points before reaching the steady state. By contrast, the C-V model’s result clearly exhibits several sharp thermal wave fronts in the temperature–time curves. Compared to the two models, the G-K model’s result predicts a much steeper initial temperature increase and removes the sharp wave fronts, which demonstrates that both the time lagging effect and the thermal nonlocal length scale are critical factors governing the transient thermal responses. To further highlight the discrepancy between the C-V and G-K heat conduction frameworks, Figure 5 presents the dimensionless temperature profiles along the thickness direction of the aluminium foam plate. The C-V model predictions show the occurrence of the infinite temperature gradient spatially, which is physically unrealistic. The G-K model, however, successfully resolves this inherent defect of the C-V model and eliminates these sharp thermal discontinuities. Consequently, ignoring the precise transient analysis in the design of aluminium foam will pose substantial risks in engineering applications.

**Figure 6:**
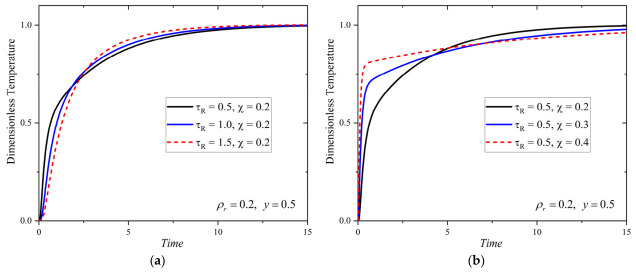
Transient dimensionless temperature with the variation in (**a**) $\tau_{R}$ and (**b**) χ under the G-K theory.

To elucidate the underlying mechanism of heat transport in aluminium foams described by the G-K model, we conduct a systematic parametric study on the effects of the thermal relaxation time and nonlocal length, with the results presented in Figure 6. Our numerical results demonstrate that both parameters illustrate a pronounced influence on the transient thermal responses, particularly during the initial heating period, while they have negligible impacts on the ultimate steady-state temperature distributions. Specifically, the thermal relaxation time introduces an effective inertia to the heat flux: a larger thermal relaxation time means that the heat flux requires a longer time to adjust to an imposed temperature gradient. This delays the establishment of a fully developed thermal field and consequently results in a slower temperature rise at early times (Figure 6a). In contrast, the nonlocal length provides an additional channel for heat transport via ballistic phonon propagation. When the nonlocal length increases, the spatial smoothing term in the G-K equation allows heat to be transported more rapidly across the plate, overcoming the delay caused by the porous structure. Hence, the early-stage temperature rises more steeply with increasing the nonlocal length (Figure 6b). These two competing mechanisms determine whether the transient thermal response is wave-like or diffusion-dominated and collectively shape the complex non-Fourier temperature history observed in the heat pulse experiments of Figure 1.

**Figure 7:**
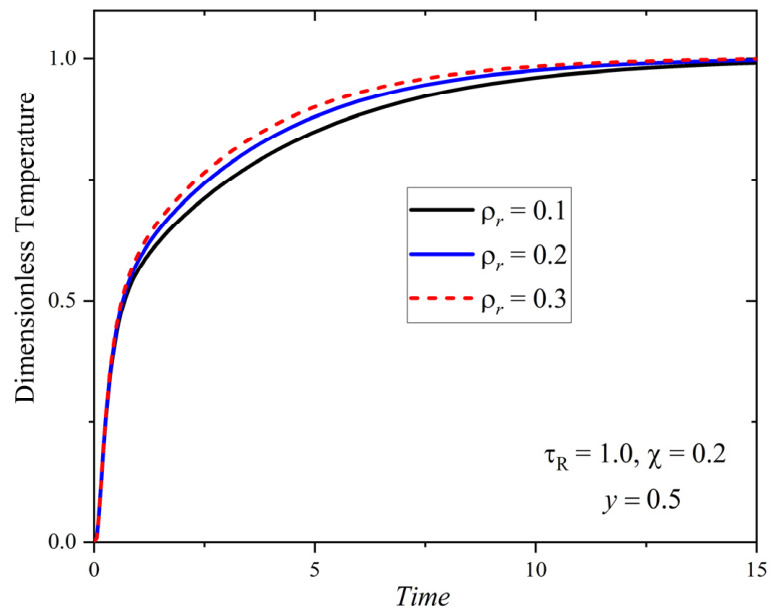
Transient dimensionless temperature with the variation in the relative density under the G-K theory.

**Figure 8:**
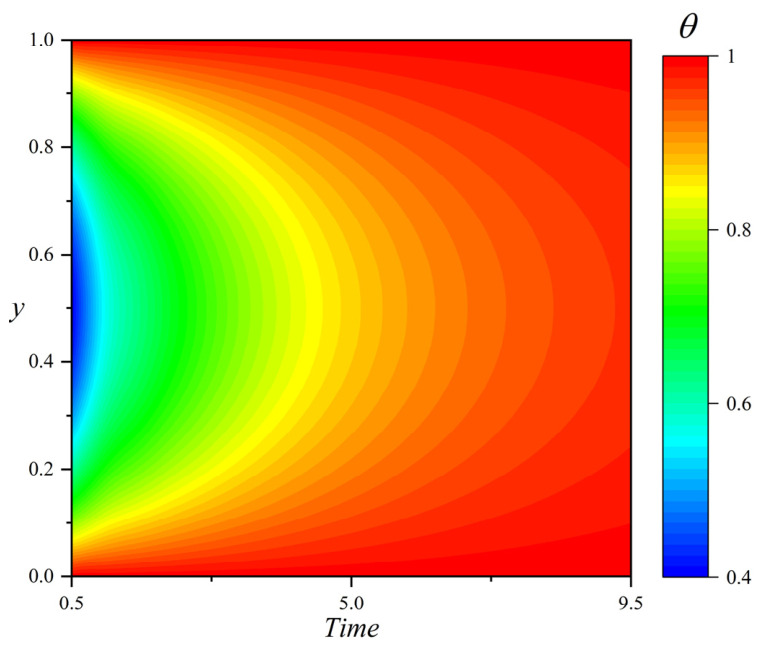
Transient dimensionless temperature evolution along the *y*-axis under the G-K theory.

As the most important structural parameter of porous metals, relative density directly determines the effective thermophysical properties of aluminium foams. To quantify its influence on transient heat conduction, Figure 7 plots the transient temperature increase at the mid-thickness point of the aluminium foam plate under various relative densities. The numerical results reveal that the relative density has almost no impact on heat transport during the initial heating phase. As the heating process continues, however, plates with larger relative densities exhibit higher thermal levels, until all samples ultimately reach the same steady-state temperature. This convergence occurs because the entire plate is eventually heated to the presumed thermal shock temperature. To provide a comprehensive visualisation of the complete spatiotemporal heat transport process, Figure 8 illustrates the thermal evolution within the aluminium plate. This two-dimensional contour plot simultaneously depicts the temporal and spatial variations in temperature, with the horizontal axis denoting time and the vertical axis corresponding to the thickness direction of the plate.

**Figure 9:**
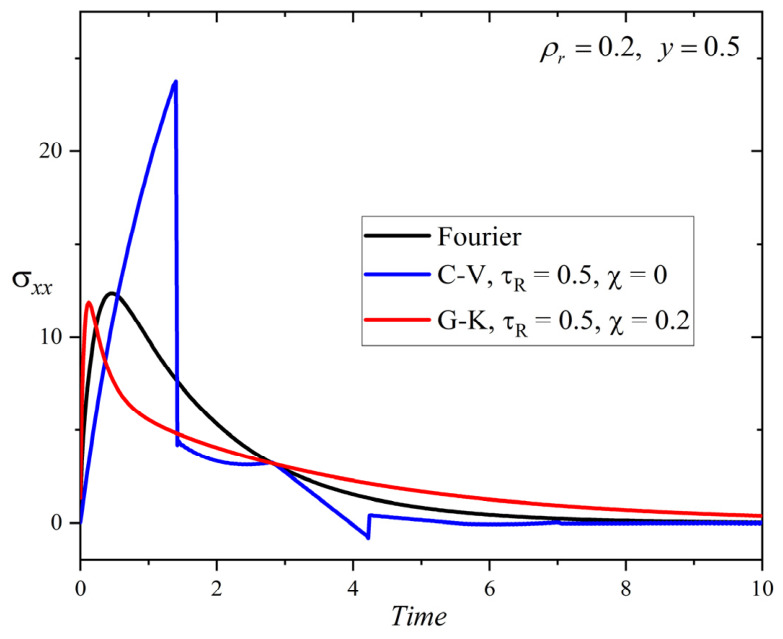
Transient thermal stress of the midpoint in the thickness direction under three heat conduction theories.

**Figure 10:**
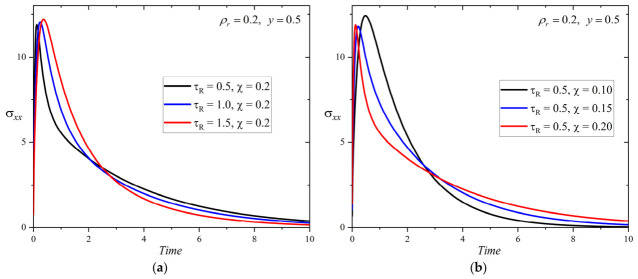
Transient thermal stresses with the variation in (**a**) $\tau_{R}$ and (**b**) χ under the G-K theory.

**Figure 11:**
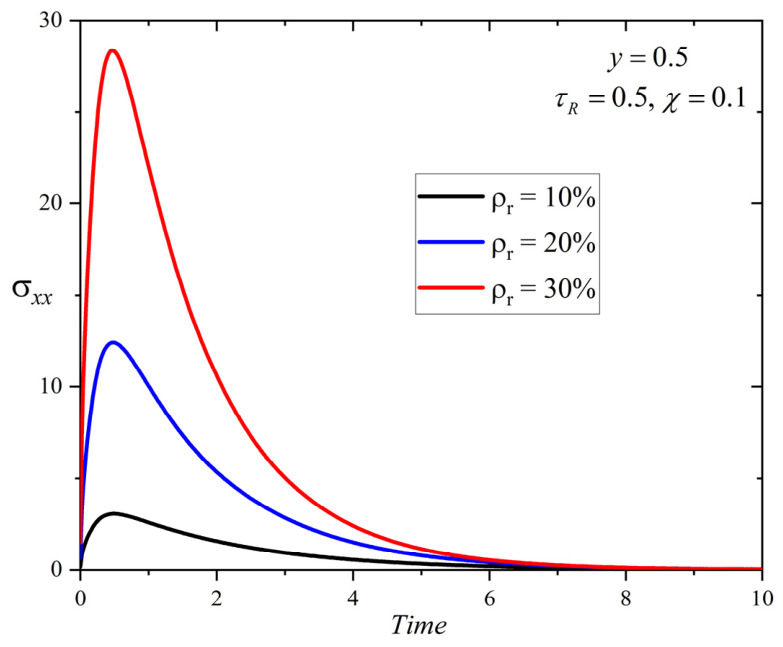
Transient thermal stress of the midpoint along the thickness direction with the variation in the relative density under the G-K theory.

Due to the thermal expansion induced deformation and the non-equilibrium thermal level distribution in the aluminium foam plate during the heating process, considerable thermal stresses are generated within the porous structure. These thermally induced stresses have a detrimental influence on the structural integrity when the external thermal shock loading is excessively high. Since different heat conduction frameworks predict distinct thermal stress behaviours in aluminium foam plates under thermal loading, Figure 9 compares the transient thermal stress at the midpoint in the thickness direction under three heat conduction theories. Similar to their effects on the temperature changes, the thermal stress time history predicted by the C-V model exhibits sharp discontinuities, which indicate unrealistic stress gradients in the time domain. Compared with Fourier’s results, the G-K model predictions suggest that the non-Fourier effect in aluminium foams is capable of elevating the thermal stress rise rate in the early heating stage and enhancing the stress levels throughout the entire period. To symmetrically quantify the effects of thermal relaxation time and nonlocal length on the thermal stress responses, Figure 10 examines the stress–time histories of the mid-thickness point. All the curves indicate that the thermal stresses always increase rapidly in the early heating stage until reaching their peak values and then decrease monotonically to the low stress levels. It is noted that increasing the thermal relaxation time leads to a slower stress rise in the early heating stage, while the nonlocal length exhibits the opposite effect and increasing the nonlocal length elevates the thermal stress rise rate. Figure 11 illustrates the influence of the porosity of aluminium foams on the stress time histories of the mid-thickness point. The relative density is noticed to be a critical factor in determining the thermal stress levels, and a larger relative density considerably increases the stress magnitudes.

**Figure 12:**
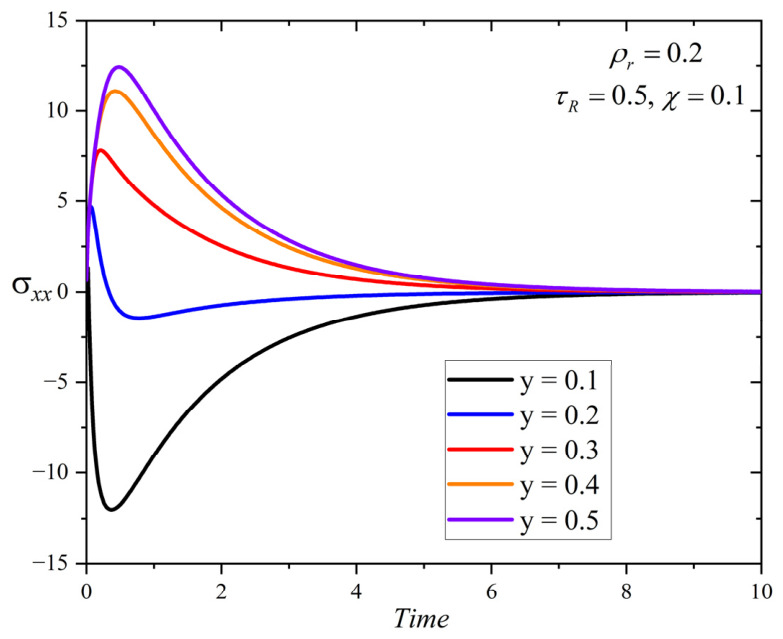
Transient thermal stress evolutions of different locations under the G-K theory.

**Figure 13:**
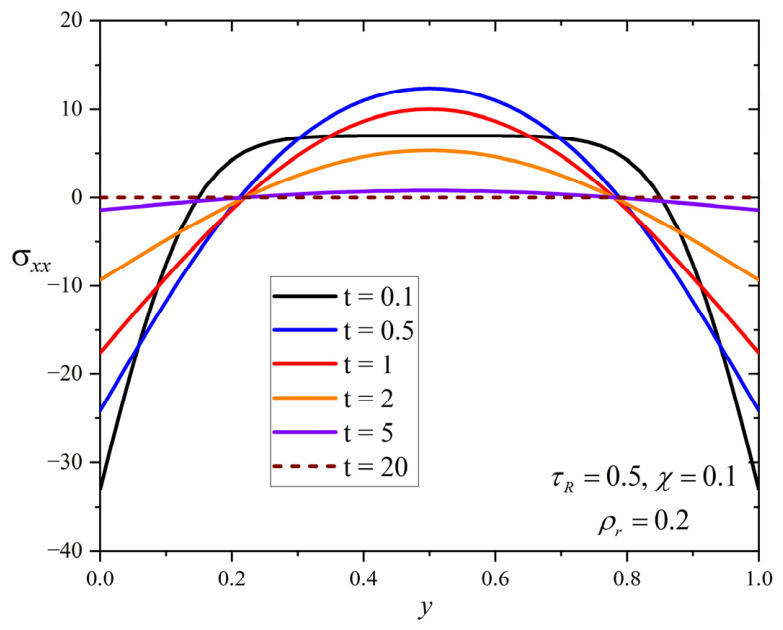
The thermal stress distributions along the thickness direction at various time instants under the G-K theory.

**Figure 14:**
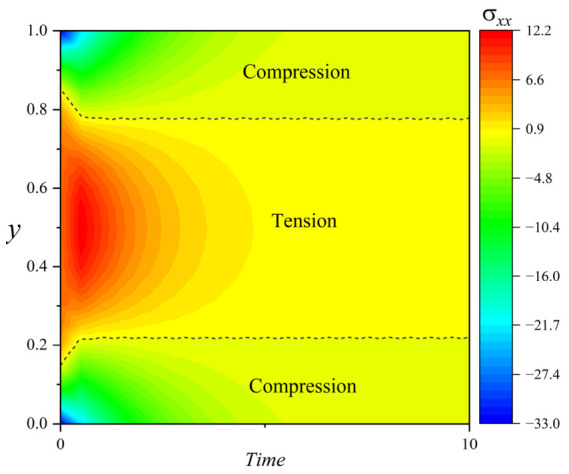
The evolution of the thermal stress along the *y*-axis under the G-K theory.

During the heating stage, different regions in the aluminium foam plate are subjected to varying temperature levels; therefore, the thermal stress responses of these regions are quite different. Due to the symmetry, Figure 12 reveals the stress time histories of several monitoring points along the thickness direction. It is noted that all the stress–time curves converge to zero eventually, which is reasonable since the foam plate is uniformly heated to the same temperature levels at last. Nevertheless, the transient responses of thermal stress show remarkable differences. The region adjacent to the heated surface experiences compressive stress, whereas the central part of the plate is under tensile stress. To obtain a comprehensive view of the thermal stress responses of aluminium foams, Figure 13 presents the thermal stress profiles at various time instants and Figure 14 plots the stress evolution within the aluminium plate. The two-dimensional contour plot simultaneously depicts the temporal and spatial variations in thermal stresses, with the horizontal axis denoting time and the vertical axis corresponding to the thickness direction of the plate. Evidently, the central region remains in the tensile stress state; therefore, the central crack problem is equivalent to the mode-I fracture problem by exerting the induced thermal stresses on the crack faces.

**Figure 15:**
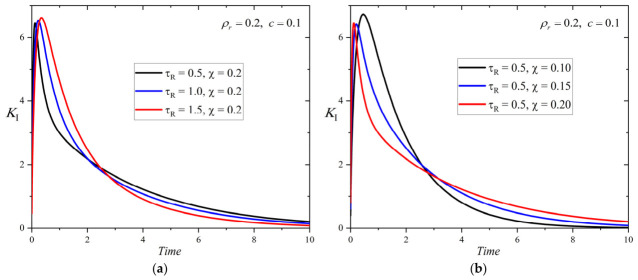
The effects of (**a**) $\tau_{R}$ and (**b**) χ on the transient SIFs under the G-K theory.

**Figure 16:**
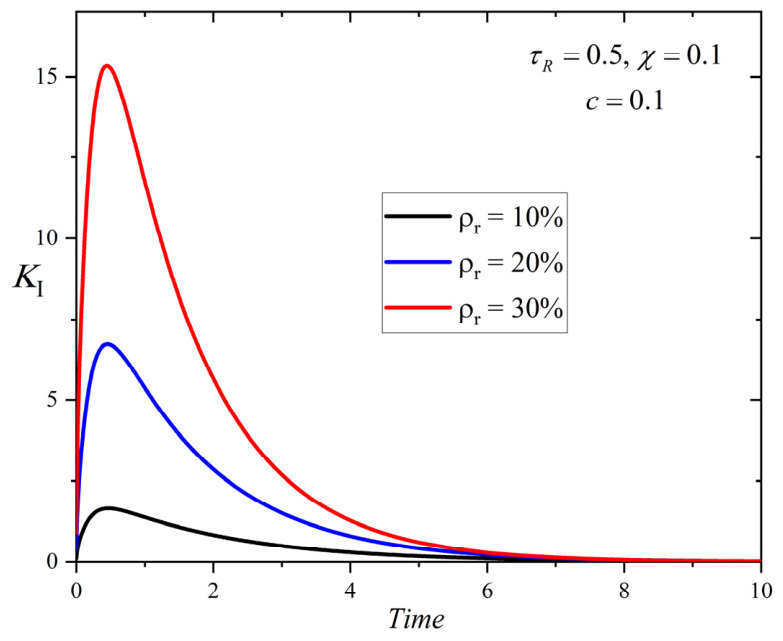
The effects of the relative density on the transient SIFs under the G-K theory.

**Figure 17:**
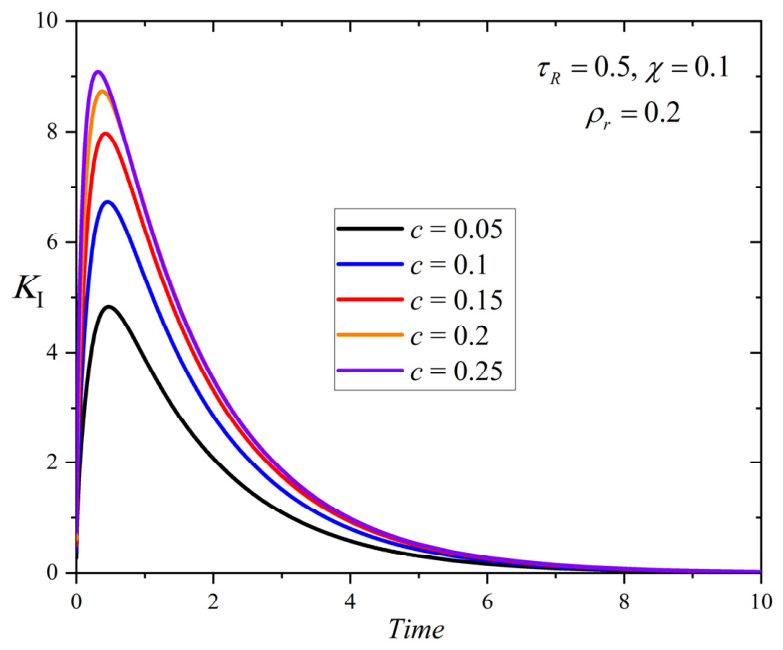
The effects of the crack length on the transient SIFs under the G-K theory.

**Figure 18:**
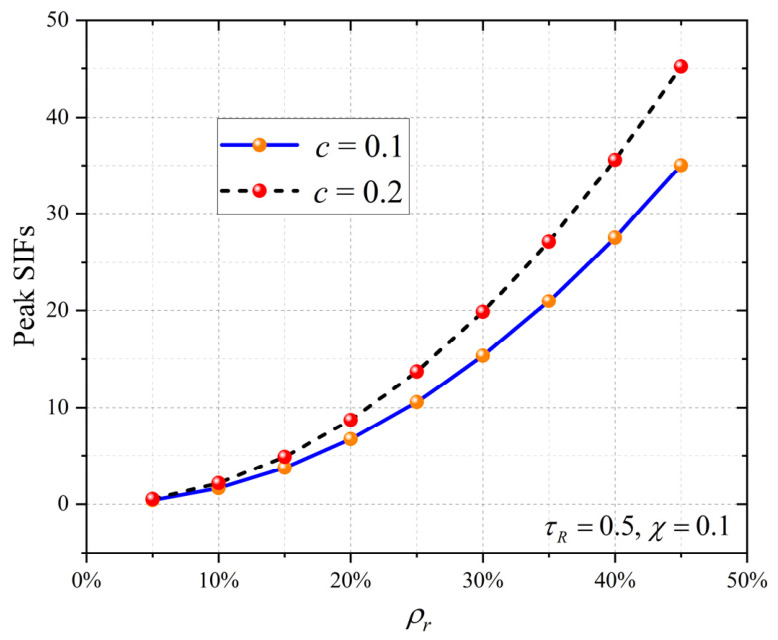
The variation in peak SIFs for various relative densities under the G-K theory.

**Figure 19:**
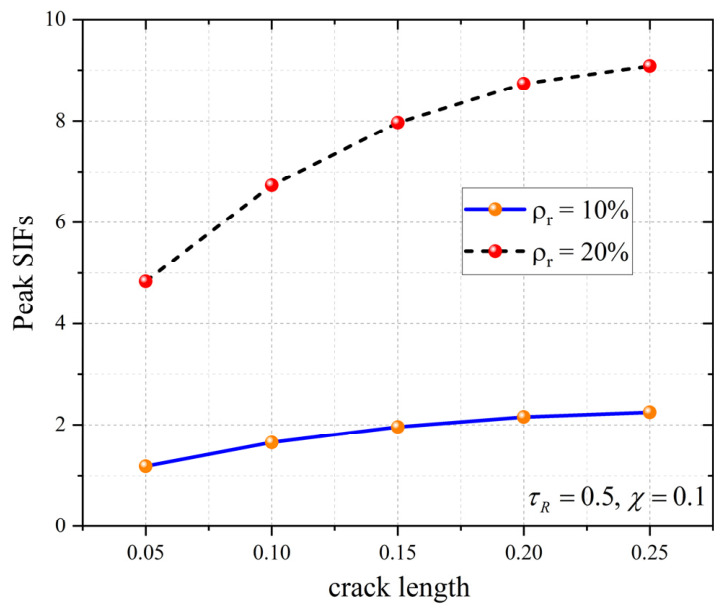
The variation in peak SIFs for various crack lengths under the G-K theory.

As a fundamental concept in linear elastic fracture mechanics, the stress intensity factor (SIF) characterises the stress field singularity at the crack tip. In the present hot thermal shock problem governed by the G-K model, the fracture behaviour is highly complex and is affected by a combination of factors, including the crack length, the thermal relaxation time, the nonlocal length parameter, and the relative density of the aluminium foams. Figure 15 presents the effects of the thermal relaxation time and nonlocal length on the transient SIFs. All the curves exhibit that the SIFs rise rapidly and reach their peak magnitudes within an extremely short period following the initiation of thermal heating, after which they gradually decrease to low values. It is observed that an increase in thermal relaxation time results in a slower growth rate of SIFs in the early heating stage, while the nonlocal length exhibits the opposite effect and the larger nonlocal length increases the rise rate of SIFs. Figure 16 examines the effect of the porosity on the SIFs of the central crack. In accordance with the influence on the thermal stress, the relative density noticeably elevates the SIF magnitudes. Subsequently, Figure 17 elucidates the effect of the crack length on the SIFs, whose results reveal that an increase in the crack length leads to higher SIFs of the central crack. Figure 18 reveals the variation in peak SIFs with various relative densities for two crack lengths, and the results demonstrate that the peak SIFs grow monotonically with the relative density, and the growth rate is higher in the longer crack. Finally, Figure 19 plots the variation in peak SIFs with various crack lengths for two relative densities, and the results suggest the peak SIFs grow monotonically with the crack length and the growth rate is higher in aluminium foams with the larger relative density.

## 5. Conclusions

Recent heat pulse experiments have demonstrated that the classical Fourier’s law fails when describing heat transport in aluminium foams at short time scales, while the G-K theory, which accounts for both thermal relaxation and nonlocal effects, performs much better in characterising their transient thermal behaviours. The present work aims to explore the non-Fourier heat transfer and thermoelastic fracture characteristics of an aluminium foam plate containing a central crack subjected to sudden thermal shocks. The weight function methodology coupled with the superposition scheme is adopted to derive the analytical solution for the mode-I stress intensity factors. A comprehensive parametric analysis is performed to evaluate the influences of thermal relaxation time, nonlocal length and relative density on the transient temperature fields, thermal stress distributions and dynamic fracture responses. Some important conclusions are:

(1) The G-K model successfully resolves the inherent defect of the C-V model and eliminates sharp thermal discontinuities.

(2) Increasing the thermal relaxation time leads to a slower initial temperature rise, whereas the nonlocal length parameter shows the opposite trend and accelerates the heating process in the early stage.

(3) The G-K model predictions suggest that the non-Fourier effect in aluminium foams is capable of elevating the thermal stress rise rate in the early heating stage and enhancing the stress levels throughout the entire period.

(4) An increase in thermal relaxation time results in a slower growth rate of SIFs in the early heating stage, while the nonlocal length exhibits the opposite effect and the larger nonlocal length increases the rise rate of SIFs.

## Figures and Tables

**Table 1:** Material constants of air and aluminium [21].

	Density	Specific Heat	Thermal Conductivity
Aluminium	$2707\;\mathrm{kg}/m^{3}$	905 J/(kg⋅K)	237 W/(mK)
Air	$1.184\;\mathrm{kg}/m^{3}$	1006 J/(kg⋅K)	0.025 W/(mK)

## Data Availability

The original contributions presented in this study are included in the article. Further inquiries can be directed to the corresponding author.
